# Changes in DNA methylation are associated with systemic lupus erythematosus flare remission and clinical subtypes

**DOI:** 10.1186/s13148-024-01792-x

**Published:** 2024-12-18

**Authors:** Mary K. Horton, Joanne Nititham, Kimberly E. Taylor, Patricia Katz, Chun Jimmie Ye, Jinoos Yazdany, Maria Dall’Era, Charlotte Hurabielle, Lisa F. Barcellos, Lindsey A. Criswell, Cristina M. Lanata

**Affiliations:** 1 Genomics of Autoimmune Rheumatic Disease Section, National Human Genome Research Institute, National Institutes of Health, Bethesda, MD USA; 2 Division of Rheumatology, Department of Medicine, University of California San Francisco, San Francisco, CA USA; 3 Bakar Computational Health Sciences Institute, University of California, San Francisco, CA USA; 4 Division of Epidemiology, University of California, Berkeley, CA USA

**Keywords:** DNA methylation, Disease subtyping, Systemic lupus erythematosus, Unsupervised machine learning, Hierarchical clustering, Longitudinal, Lupus flare, Lupus remission

## Abstract

**Background:**

Systemic lupus erythematosus (SLE) has numerous symptoms across organs and an unpredictable flare-remittance pattern. This has made it challenging to understand drivers of long-term SLE outcomes. Our objective was to identify whether changes in DNA methylation over time, in an actively flaring SLE cohort, were associated with remission and whether these changes meaningfully subtype SLE patients.

**Methods:**

Fifty-nine multi-ethnic SLE patients had clinical visits and DNA methylation profiles at a flare and approximately 3 months later. Methylation was measured using the Illumina EPIC array. We identified sites where methylation change between visits was associated with remission at the follow-up visit using *limma* package and a *time x remission* interaction term. Models adjusted for batch, age at diagnosis, time between visits, age at flare, sex, medications, and cell-type proportions. Separately, a paired T-test identified Bonferroni significant methylation sites with ≥ 3% change between visits (*n* = 546). Methylation changes at these sites were used for unsupervised consensus hierarchical clustering. Associations between clusters and patient features were assessed.

**Results:**

Nineteen patients fully remitted at the follow-up visit. For 1,953 CpG sites, methylation changed differently for remitters vs. non-remitters (Bonferroni *p* < 0.05). Nearly half were within genes regulated by interferon. The largest effect was at cg22873177; on average, remitters had 23% decreased methylation between visits while non-remitters had no change. Three SLE patient clusters were identified using methylation differences agnostic of clinical outcomes. All Cluster 1 subjects (*n* = 12) experienced complete flare remission, despite similar baseline disease activity scores, medications, and demographics as other clusters. Methylation changes at six CpG sites, including within immune-related *CD45* and *IFI* genes, were particularly distinct for each cluster, suggesting these may be good candidates for stratifying patients in the future.

**Conclusions:**

Changes in DNA methylation during active SLE were associated with remission status and identified subgroups of SLE patients with several distinct clinical and biological characteristics. DNA methylation patterns might help inform SLE subtypes, leading to targeted therapies based on relevant underlying biological pathways.

**Supplementary Information:**

The online version contains supplementary material available at 10.1186/s13148-024-01792-x.

## Introduction

Systemic lupus erythematosus (SLE) is a complex autoimmune disease that follows a relapsing (flare)-remitting pattern with systemic symptoms that vary greatly from person to person. The disease activity of SLE involves a dynamic interplay between molecular processes, particularly interferon pathways. How exactly this relates to flares, remission, and organ damage are largely unknown. Importantly, clinical characteristics are not strongly correlated with response to treatment, disease activity, or future prognosis. Given the diversity of symptoms and flare-remittance patterns of illness, it has been challenging to predict long-term SLE outcomes.

Patterns of DNA methylation (DNAm) have emerged as potential mechanisms for and biomarkers of SLE risk, disease activity, and heterogeneity [1–7]. Identifying differences in DNAm by SLE phenotypes or flare remission status might also illuminate mechanistic pathways responsible for SLE heterogeneity. Recently, much effort has been devoted to identifying SLE patient subtypes to better characterize disease heterogeneity. These efforts have primarily been based on bulk and single cell RNA sequencing, clinical symptoms, autoantibodies, and gene sequencing [8–17]. The strong involvement of type I interferon signaling has consistently emerged as one SLE patient subtype, while others have varied. Despite this consistency and the great need to stratify SLE patients, these studies have predominantly been cross sectional with participants not necessarily experiencing active disease flares. To the best of our knowledge, no studies have subtyped SLE patients based on changes in DNAm at different points of disease activity. Additionally, there has been limited inclusion of non-white participants in efforts to subtype SLE, a significant limitation given the worse disease outcomes and higher incidence among African American, Hispanic, and Asian individuals [18, 19].

In this study, we utilized 59 multi-ethnic SLE patients recruited during an active flare who have DNAm profiles at baseline flare and approximately three months later. Our objectives were to: (1) identify whether changes in DNAm between visits were associated with complete remission status at follow-up, and (2) perform unsupervised clustering of changes in DNAm to identify clinically or biologically relevant SLE subtypes.

## Methods

### Study subjects and design

Individuals experiencing an active SLE flare were recruited to the California Lupus Epidemiology Study (CLUES) Flare Cohort during routine clinical care at the University of California, San Francisco (UCSF). SLE diagnoses were confirmed by study physicians based upon one of the following definitions: (a) meeting ≥ 4 of the 11 American College of Rheumatology (ACR) revised criteria for the classification of SLE as defined in 1982 and updated in 1997, (b) meeting 3 of the 11 ACR criteria plus a documented rheumatologist’s diagnosis of SLE, or (c) a confirmed diagnosis of lupus nephritis, defined as fulfilling the ACR renal classification criterion (> 0.5 g of proteinuria per day or 3 + protein on urine dipstick analysis) or having evidence of lupus nephritis on kidney biopsy. A flare was established by the treating physician and characterized by the SELENA Systemic Lupus Erythematosus Disease Activity Index (SLEDAI) [20]. A SLEDAI ≥ 3 was required for inclusion and physician determination that a flare was significant enough to warrant a treatment change. Approximately three months later, participants returned to the clinic as part of routine clinical care. Individuals with blood samples and complete clinical data at each visit were included in our sample. This resulted in a total of 122 paired samples (61 individuals).

This study was approved by the Institutional Review Board of UCSF. All participants signed a written informed consent to participate.

### DNA methylation data

Genome-wide DNAm was measured using the Illumina Infinium MethylationEPIC BeadChip v1.0 from DNA extracted from whole blood at each visit. Both samples from an individual were run on the same plate/array. DNAm data was processed using the *minfi* package in R version 4.3.3 [21, 22]. Signal intensities were background subtracted using the noob function and quantile normalized. We excluded both paired samples from individuals if at least one of their samples had ≥ 5% detection *p* < 0.01, self-reported sex did not match sex predicted from DNAm (*n* = 1), or if paired samples had mismatched genotypes detectable from the methylation array (*n* = 1). CpG sites were removed if ≥ 5% detection *p* < 0.01, overlapped with annotated single nucleotide polymorphisms (SNPs) from any ancestral population in the single base extension or CpG, overlapped with previously identified cross-reactive probes, or were on sex chromosomes [23, 24]. This resulted in 606,337 CpG sites across 118 samples for analyses.

Global cell-type proportions for 12 leukocyte subtypes (neutrophils, eosinophils, basophils, monocytes, naive and memory B cells, naive and memory CD4 + and CD8 + T-cells, natural killer, and T regulatory cells) were estimated at each visit using *estimateCellCounts2()* with EPIC IDOL-Ext library CpGs in the *FlowSorted.Blood.EPIC* package in *R* [25]. Input DNAm data were normalized using *preprocessNoob()*.

### Clinical data

Clinic visits included the collection and review of medical records, a SLE history and physical examination conducted by a physician specializing in SLE, and completion of a structured interview by an experienced research assistant. Data collected included sex, self-reported race, Hispanic ethnicity, age, and SELENA SLEDAI components and score at each visit. Medication assessment included whether individuals were on the following at the time of each blood draw: prednisone, cyclophosphamide, mycophenolate mofetil or mycophenolic acid, rituximab, belimumab, azathioprine, cyclosporine, tacrolimus, hydroxychloroquine, methotrexate, and solumedrol. One person had missing medication for the follow-up visit so their medications were imputed with the baseline visit medications. Multiple correspondence analysis (MCA), similar to principle component analysis but for categorical variables, was conducted separately at each time point on binary medication variables to reduce the dimensionality of the medication data using the *FactoMineR* *R* package [26]. The *EpiSmokEr* *R* package was used to predict smoking status (current, past, never) using DNA methylation data [27].

### Statistical analyses

#### Differential methylation position analyses for remission

Our primary objective was to identify CpG sites where changes in DNAm between visits were associated with complete remission status at follow-up. Because we were limited to two timepoints and without additional longitudinal clinical data, it was not possible to determine if patients who only had serologic activity (presence of double-stranded DNA antibodies and/or low complement) were clinically quiescent. Therefore, we conservatively defined “remitter” as a patient having a SELENA SLEDAI score = 0 at the follow-up visit. For each CpG site that reached quality control thresholds, we used a linear model with a *time x remission* interaction term in *limma* *R* package [28]. Time represented baseline flare (time = 0) or follow-up (time = 1) visits. The paired design was accounted for using the *duplicatecorrelation()* function. DNAm m-values were used to obtain respective p-values while DNAm beta-values were reported for coefficient interpretation [29]. Interaction coefficients were interpreted as the average difference in percent change in DNAm between baseline and follow-up comparing the remitters to non-remitters at each CpG site. The primary model (Model 1) included plate batch, sex, diagnosis age, baseline age, and days between visits. CpG sites with *p* < 0.05/606,337 were considered Bonferroni significant. For sites that reached this threshold and had an interaction coefficient absolute value ≥ 0.1, we additionally tested for the effect of medications (Model 2) and cell -type proportions (Model 3) on observed associations. Model 2 included Model 1 covariates plus the first medication MCA component. Additional components were not included because they were not associated (*p* < 0.05) with remission status and/or DNAm principal components in bivariate logistic or linear regression models. Model 3 included all covariates from Models 1 and 2 plus seven estimated cell-type proportions for SLE-relevant cell types, *e.g.,* neutrophils, naïve and memory CD4 + T-cells, naïve and memory CD8 + T-cells, and naïve and memory B cells. Only one patient was predicted to be a current smoker at baseline and none were predicted to be current smokers at follow-up. Because we were interested in whether changes in DNA methylation were associated with remission status, we wouldn’t expect unchanged smoking behaviors to affect change in methylation. For this reason, smoking status was not included as a covariate.

#### Unsupervised clustering of DNA methylation difference data

We sought to identify whether changes in DNAm between visits might be able to subtype patients into clinically or biologically relevant subgroups. We used a paired t-test in *limma* to identify sites where DNAm changed between visits, agnostic of clinical outcomes. DNAm m-values were used to obtain respective p-values while DNAm beta-values were used to obtain interpretable coefficients. CpGs with false discover rate (FDR) *q* < 0.05 and ≥ 0.03 DNAm change between visits were used for clustering (“cluster input CpGs”). The standardized difference in DNAm beta-values between baseline flare and follow-up visits were input into the consensus hierarchical clustering algorithm using *ConsensusClusterPlus* *R* package [30]. Pearson distance with average linkage was used. Agglomerative hierarchical clustering was repeated 1,000 times, with 80% of CpG sites and 80% of participants resampled per iteration. The optimal cluster number was determined based on the following criteria: a relatively low-variation coefficient within the cluster, relatively high consistency, and no obviously increased area under the cumulative distribution function curve.

#### Associations with SLE methylation subtypes

To determine whether SLE patient clusters identified from DNAm data had distinct demographic or clinical characteristics, we used chi-square and ANOVA tests. Demographics included sex, self-reported race, and Hispanic ethnicity. Clinical features, at both visits when relevant, included age at visit, diagnosis age, SELENA SLEDAI total score and components, remission status, and medications. Estimated proportions of 12 cell types derived from DNAm data at each visit were also assessed.

To determine which cluster input CpG sites were most strongly contributing to cluster assignments, we first used an ANOVA to identify DNAm changes that were different between any clusters. For CpGs with ANOVA *p* < 0.05/# input CpGs, we used a bivariate linear regression model to estimate associations between DNAm differences at each cluster input CpG (outcome) and cluster (predictor). Cluster was represented as a categorical variable with one cluster used as a common reference category. A CpG site was considered significant for a particular cluster if the respective cluster category regression term had *p* < 0.05/# cluster input CpGs significant from ANOVA.

#### Pathway analyses

Pathway analyses were conducted to test for enrichment of Reactome terms among several sets of CpG sites from analyses. This was conducted using the *methylgometh* function in the *methylGSA* *R* package [31]. Pathways with FDR *q* < 0.05 were considered significantly enriched. Because of the strong involvement of interferon signaling in SLE, we annotated CpG sites according to whether they were within interferon regulated genes (IRGs) (identified in experiments when cells or organisms were treated with an interferon) using the Interferome Database v2.01 [32].

## Results

### Characteristics of participants

Nineteen participants fully remitted by the follow-up visit (Table 1). On average, remitters were five years older than non-remitters and diagnosed in their mid-30s, compared to mid-20s. Within this multi-ethnic cohort, remitters and non-remitters did not have significantly different sex, race, or ethnicity. SLEDAI at the baseline flare were similar among remitters and non-remitters (mean = 11.4 (standard deviation (sd) = 5.7) and 11.5 (sd = 5.9), respectively). At the follow-up visit, non-remitters had a mean SLEDAI = 5.7 (sd = 4.1). At baseline, both groups had similar presence of SLEDAI proteinuria (42% each), an indicator of lupus nephritis, a severe manifestation of SLE. All SLEDAI components and estimated cell-type proportions were similar among remitters and non-remitters, except for CD8 + naïve T-cells at baseline and follow-up and natural killer cells at baseline (Supplementary Table 1). Patients were not treatment-naïve at baseline and medication use at the baseline and follow-up visits were similar between remitters and non-remitters (Supplementary Table 1).

**Table 1:** Clinical and demographic features of 59 systemic lupus erythematosus participants by remission status at follow-up visit

	Remission status		
	Yes	No	*P-value*
N	19	40	
Female, n (%)	18 (94.74)	34 (85.00)	0.52
Self-reported race, n (%)			0.79
White	1 (5.26)	6 (15.00)	
Black	3 (15.79)	6 (15.00)	
Asian	7 (36.84)	11 (27.50)	
Other	0 (0.00)	2 (5.00)	
Multiple races^a^	1 (5.26)	2 (5.00)	
Not reported^b^	7 (36.84)	13 (32.50)	
Hispanic, n (%)	7 (36.84)	15 (37.50)	1.00
Age at diagnosis (years), mean (sd)	35.68 (16.34)	27.05 (10.57)	0.02
Age at baseline flare visit (years), mean (sd)	40.68 (13.64)	35.88 (12.20)	0.18
Days between visits, median [IQR]	96.00 [57.00, 267.50]	78.50 [48.50, 163.25]	0.48
SELENA SLEDAI, mean (sd)			
Baseline	11.42 (5.65)	11.53 (5.85)	0.95
Follow-up	0.00 (0.00)	5.72 (4.08)	< 0.001
SLEDAI Proteinuria, n (%)			
Baseline	8 (42.11)	17 (42.50)	1.00
Follow-up	0 (0.00)	6 (15.00)	0.19
Medications at follow-up (yes/no), n (%)			
Prednisone	15 (78.95)	31 (77.50)	1.00
Hydroxychloroquine	17 (89.47)	32 (80.00)	0.59
Mycophenolate	7 (36.84)	15 (37.50)	1.00
Azathioprine	0 (0.00)	7 (17.50)	0.13
Belimumab	1 (5.26)	5 (12.50)	0.69
Cyclophosphamide	1 (5.26)	3 (7.50)	1.00
Solumedrol	1 (5.26)	0 (0.00)	0.70
Rituximab	1 (5.26)	3 (7.50)	1.00
Tacrolimus	0 (0.00)	2 (5.00)	0.82
Methotrexate	3 (15.79)	5 (12.50)	1.00

Remission defined as SLEDAI = 0 at follow-up visit

IQR, interquartile range; sd, standard deviation; SLE, Systemic Lupus Erythematosus; SELENA SLEDAI, SLE Disease Activity Index—the Safety of Estrogens in Lupus Erythematosus: National Assessment trial

^a^Additional race categories not solely indicated by participants included American Indian and Pacific Islander

^b^Majority of participants not reporting race identified as Hispanic

### Changes in DNA methylation among active SLE patients were associated with remission status

**Fig. 1:**
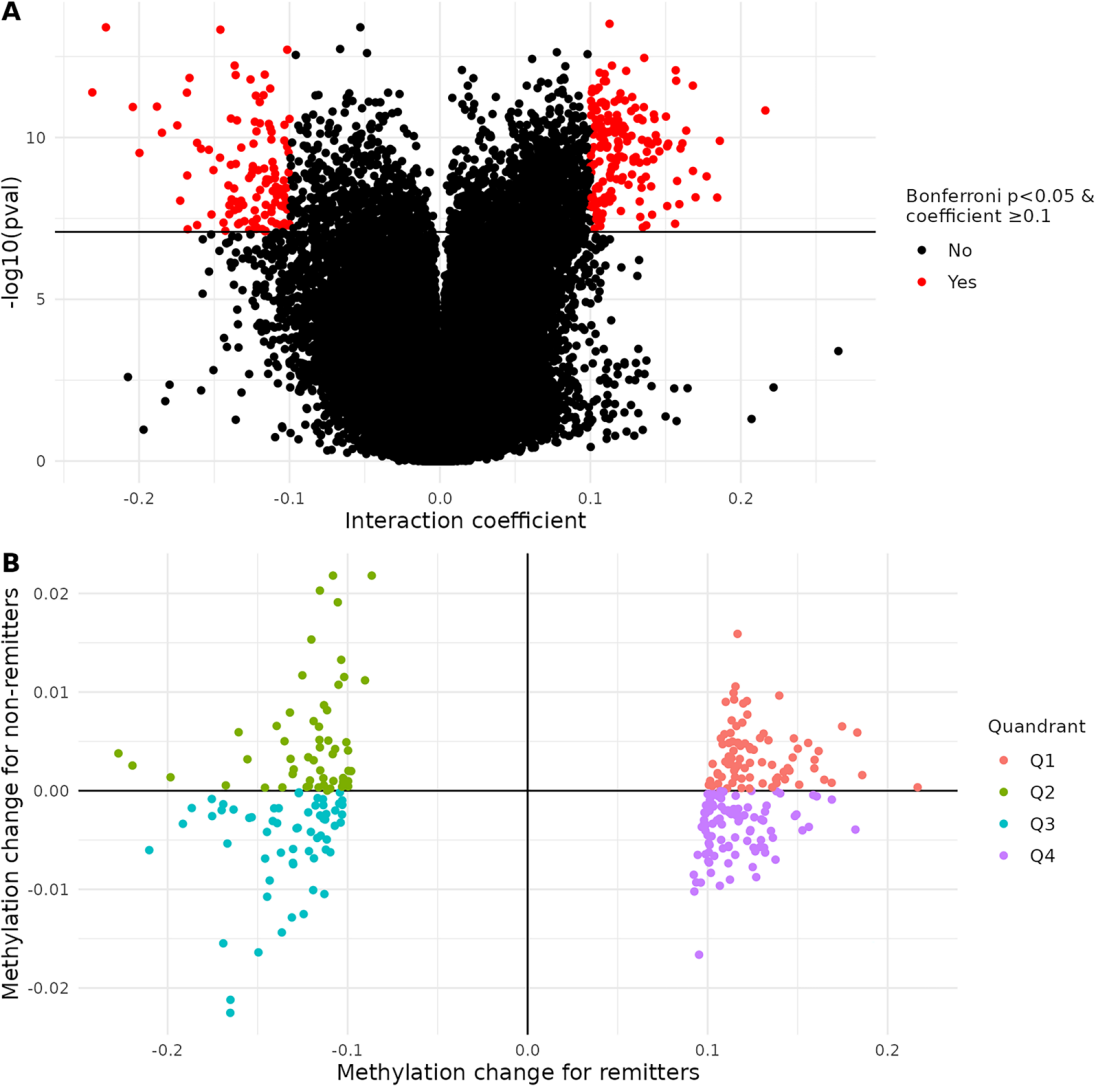
Changes in DNA methylation among active SLE patients were associated with remission status. **A** Differential methylation change between baseline flare and follow-up visits by remission status, with Model 1 covariates (in red, Bonferroni *p* < 0.05 and coefficient absolute value ≥ 0.1). Black line indicates Bonferroni significance. **B** Among CpGs with Bonferroni *p* < 0.05 and coefficient absolute value ≥ 0.1, average methylation change from baseline flare to follow-up visit for remitters (x-axis) and non-remitters (y-axis). Coefficients shown were from Model 1. Quadrants one (red) and three (blue) represent methylation changes in the same direction of effect (hypo- or hyper-methylation) for remitters and non-remitters. Quadrants 2 (green) and four (purple) represent opposite direction of methylation change between groups

**Fig. 2:**
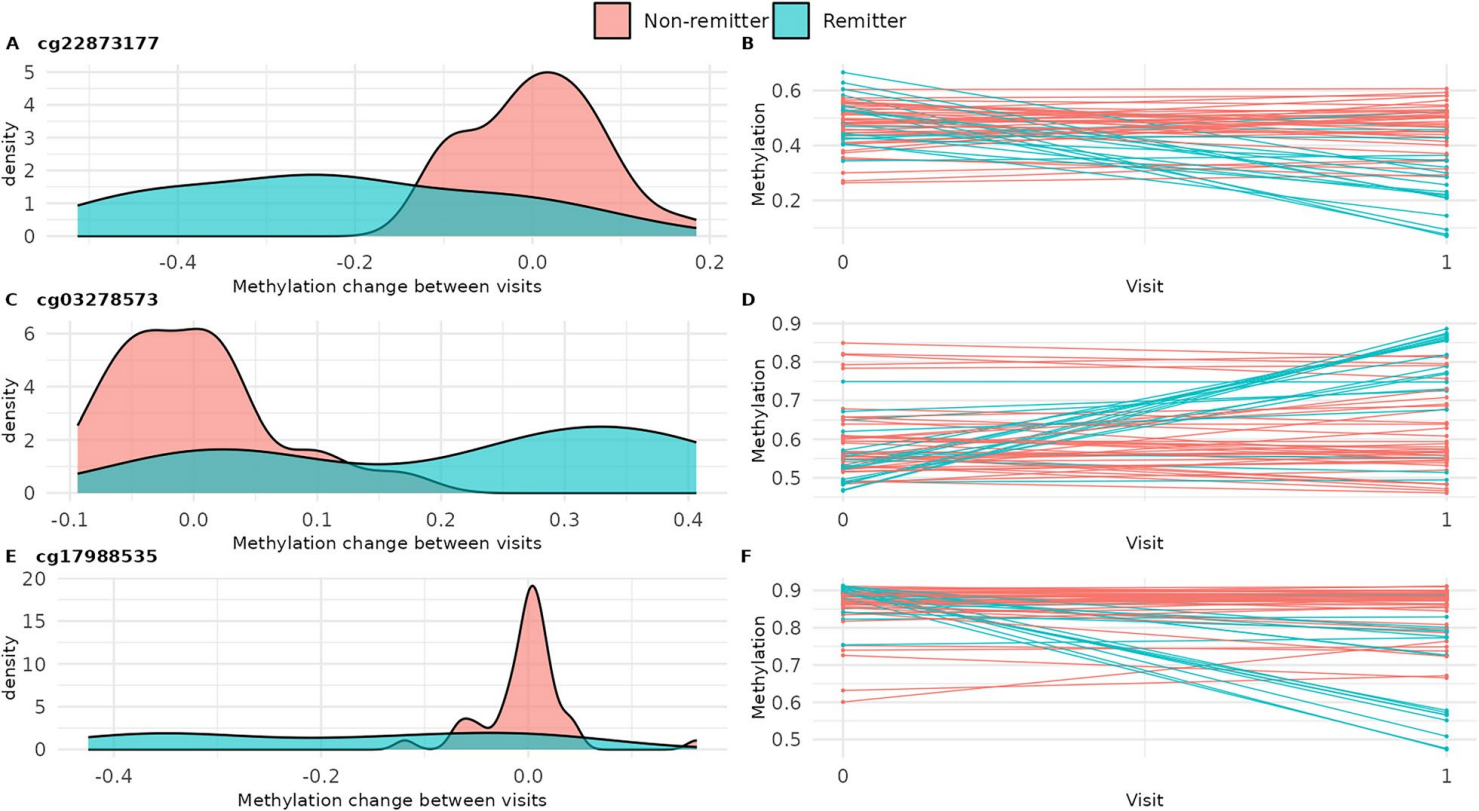
Distributions of three genome-wide significant methylation sites. Density plot of change in methylation between visits and methylation trajectories for remitters and non-remitters for cg22873177 (**a** and **b**), cg03278573 (**c** and **d**), and cg17988535 (**e** and **f**)

Our primary objective was to identify CpG sites where DNAm changed between baseline flare and follow-up visits differently depending on remission status. After adjusting for batch, sex, diagnosis age, baseline age, and days between visits (Model 1), we identified 1,953 significant CpG sites (Fig. 1A). Of these, 291 had absolute value ≥ 0.10 (provided in Supplementary Table 2 and subsequently described). DNAm at these sites changed little between visits for non-remitters (mean absolute change of 0.00) but more for remitters (mean absolute change of 0.12) (Fig. 1B). For remitters, approximately 60% (*n* = 176) of these sites had higher DNAm at the follow-up visit compared to the baseline flare (Fig. 1B); 131 were annotated to IRGs (Supplementary Table 2) [33]. These CpGs were not significantly enriched in pathways at FDR *q* < 0.05 (Supplementary Table 3). The 25 CpGs with the largest absolute interaction coefficient effect size are shown in Table 2. After adjusting for medications (Model 2) and cell-type proportions (Model 3), these 25 results remained Bonferroni significant and interaction effect sizes did not appreciably change (Supplementary 2). For three of these sites within immune-related genes, distributions of DNAm change and patients’ DNAm trajectories between visits are shown in Fig. 2. Site cg22873177, upstream of *URB2,* had the largest effect (interaction coefficient − 0.23, 95% confidence interval (CI): − 0.29, − 0.17). At this site, DNAm decreased 23%, on average, from baseline flare to follow-up for remitters and did not change for non-remitters (Fig. 2A and B, Supplementary Table 2). For the second, cg03278573, within the body of death associated protein (*DAP*), DNAm increased 22%, on average, from baseline flare to follow-up for remitters and was unchanged for non-remitters (Fig. 2C and D). For the third, cg17988535, within the body of *RASA3* (involved in T-cell homeostasis), DNAm decreased 19%, on average, from baseline flare to follow-up for remitters and was unchanged for non-remitters (Fig. 2E and F).

**Table 2:** Difference in DNA methylation change from baseline flare to follow-up visit comparing remitters to non-remitters (*visit x remission* interaction) for 25 CpGs genome-wide significant from Model 1 with largest interaction coefficient

						Model 1		
Names	chr	pos	Gene	Group	Gene’s relevance to immune function, SLE, or autoimmunity	Coef	95% CI	P-value
cg22873177	chr1	229,761,584	***URB2; TAF5L***	TSS1500; 5’UTR	Expression associated with cell cycle and TGF beta, ERBB, RIG I-like receptor, and P53 signaling pathways. PMID: 37033651	− 0.23	− 0.29, − 0.17	4.04E-12
cg14543959	chr3	113,557,658	***GRAMD1C***	TSS200	Expression associated with IL-6 levels. PMID: 25311648	− 0.22	− 0.28, − 0.17	3.94E-14
cg03278573	chr5	10,741,861	***DAP***	Body	Multiple eQTLs downregulate its transcription in immune cells; expression associated with higher autoantibody titers. PMID: 33213505	0.22	0.16, 0.28	1.46E-11
cg04038932	chr9	135,286,214	*C9orf171*	Body		− 0.20	− 0.26, − 0.15	1.14E-11
cg00102561	chr9	138,799,260	***CAMSAP1***	TSS1500	Expression associated with cell cycle and TGF beta, ERBB, and T-cell receptor signaling pathways. PMID: 36212130	− 0.20	− 0.26, − 0.14	3.00E-10
cg23945273	chr4	120,987,944	***MAD2L1***	5’UTR; 1stExon		− 0.19	− 0.24, − 0.13	1.11E-11
cg17988535	chr13	114,808,856	*RASA3*	Body	T-cell homeostasis; promotes pathogenic T helper 17 cell generation. PMID: 37545505, 30446383	− 0.19	− 0.24, − 0.13	7.14E-11
cg08323960	chr10	45,684,508				0.19	0.13, 0.24	1.27E-10
cg24566341	chr7	98,909,383				− 0.18	− 0.22, − 0.13	4.22E-11
cg22615071	chr3	156,432,778				0.18	0.12, 0.23	1.59E-09
cg10369197	chr17	70,815,226	*SLC39A1*	Body	Expression associated with cell cycle and TGF beta, NOD-like receptor, and MAPK signaling pathways. PMID: 35211427	0.18	0.12, 0.24	7.18E-09
cg09241617	chr16	7,473,688	*RBFOX1*	Body	Implicated in SLE GWAS. PMID: 28246883	− 0.17	− 0.21, − 0.13	1.44E-12
cg26118675	chr2	96,256,841	*TRIM43*	TSS1500	Antiviral defense mechanisms. PMID: 30420784	0.17	0.13, 0.21	2.49E-12
cg08399134	chr6	126,344,877	*TRMT11*	Body	T-cell proliferation; associated with lupus nephritis. PMID: 36168063, 34923866	− 0.17	− 0.21, − 0.12	4.12E-12
cg17264064	chr18	47,322,166	*ACAA2*	Body	Downregulated in T-cells of lupus-prone mice. PMID: 37216123	0.17	0.12, 0.22	1.08E-09
cg11986223	chr7	26,824,073	***SKAP2***	Body	myeloid cell activation and migration. PMID: 34172489	− 0.17	− 0.22, − 0.12	1.49E-09
cg12615557	chr1	109,046,245				0.17	0.12, 0.22	7.06E-09
cg24600355	chr20	34,329,943	*RBM39*	5’UTR; 1stExon; Body	Splicing factor highly expressed in immune cells such as CD4^+^ and CD8^+^ T cells. PMID: 34389703	− 0.17	− 0.23, − 0.12	8.96E-09
cg06089892	chr2	128,101,198	***MAP3K2***	TSS1500	Kinase dysregulated in SLE. PMID: 36309313	− 0.17	− 0.22, − 0.11	6.86E-08
cg00470768	chr15	41,285,147	***INO80***	Body	Chromatin remodeling; cell fate. PMID: 34139016	0.16	0.12, 0.21	6.13E-11
cg00350932	chr2	86,335,912	***PTCD3***	Body		− 0.16	− 0.21, − 0.12	1.46E-10
cg06176124	chr3	105,588,009	***CBLB***	TSS200	May contribute to the deregulated activation of T lymphocytes observed in SLE. PMID: 21558139	0.16	0.12, 0.20	1.51E-10
cg05062679	chr10	94,000,186	***CPEB3***	5'UTR	Associated with immune biomarkers and infiltrating immune cell types. PMID: 38476610	0.16	0.12, 0.20	2.20E-10
cg12981595	chr17	39,254,427	***KRTAP4-8***	TSS200		0.16	0.11, 0.21	1.15E-08
cg13529755	chr1	149,138,398				− 0.16	− 0.22, − 0.10	5.09E-08

chr, chromosome; CI, confidence interval; coef, interaction coefficient; pos, hg37 position; UTR, untranslated region; SLE, systemic lupus erythematosus; TSS, transcription start site

Bolded genes are interferon regulated genes

### Within-subject changes in DNA methylation stratify SLE patients into three clinically relevant subgroups

**Fig. 3:**
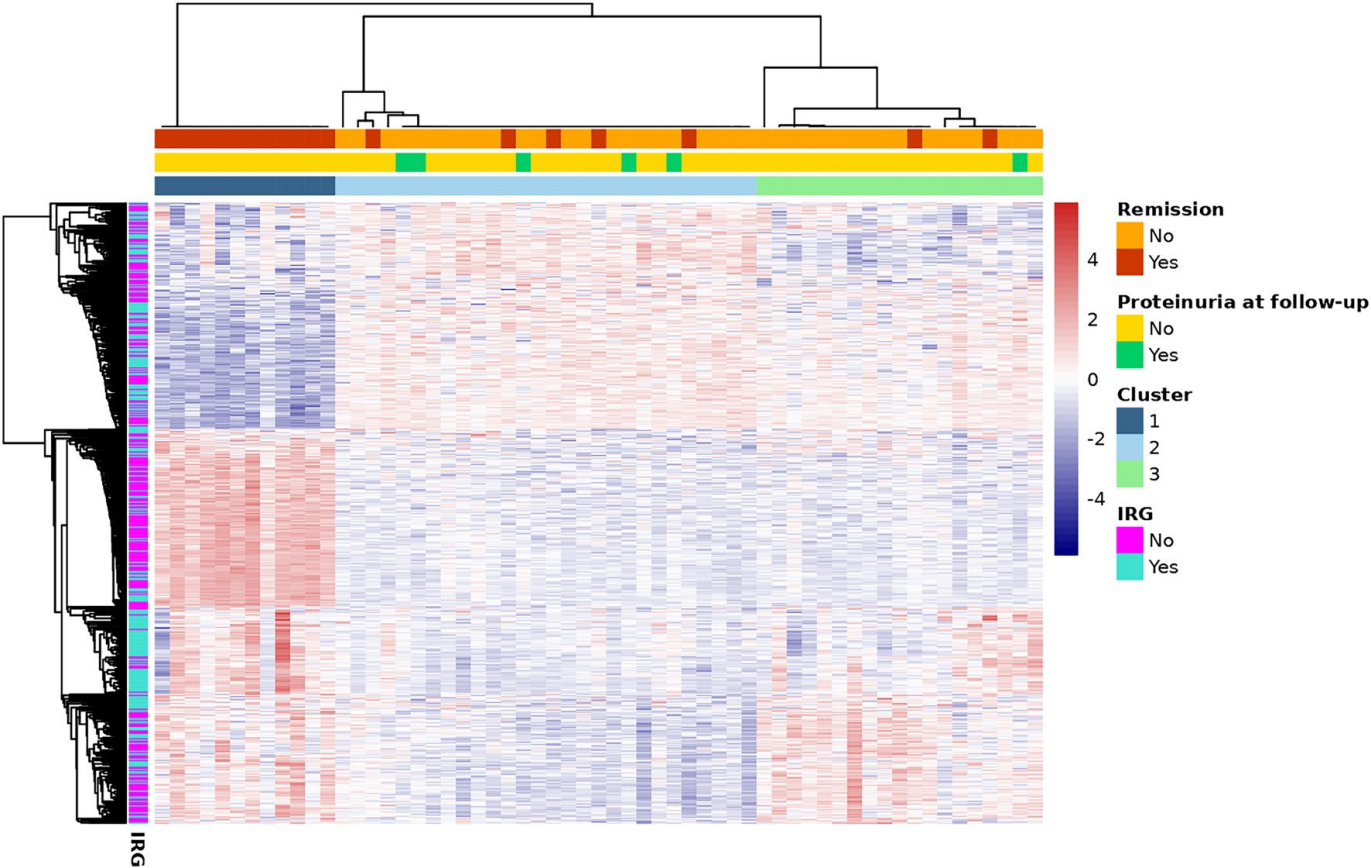
Three SLE clusters were identified from methylation changes over time from 546 cluster input CpGs. Rows represent CpG sites and were annotated as being within an interferon regulated gene (IRG). Red represents increased methylation from baseline to follow-up while blue denotes decreased. Columns represent participants and were annotated with cluster, remission status, and SLEDAI proteinuria at follow-up visit

**Fig. 4:**
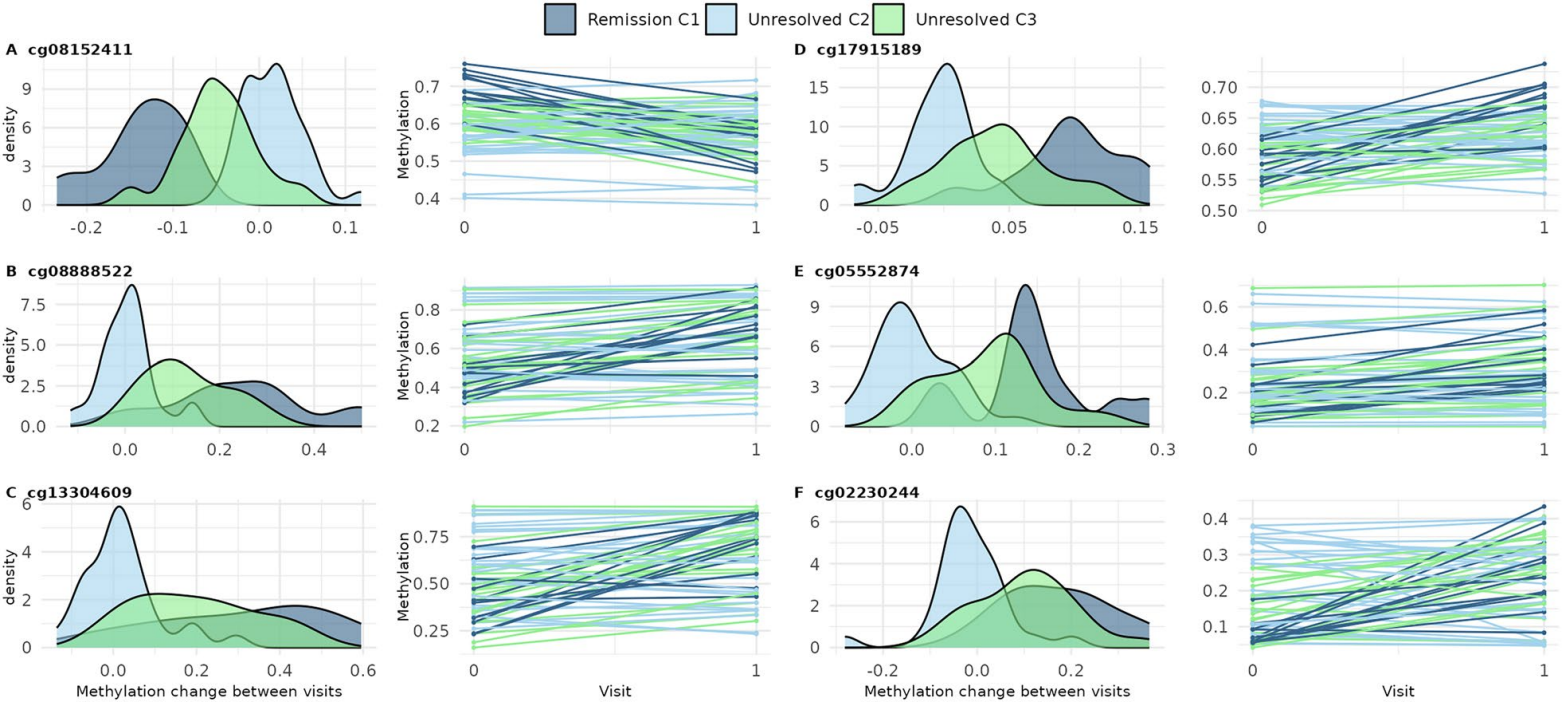
Six CpGs had different methylation change over time depending on methylation cluster. Density plot of change in methylation between visits and methylation trajectories for remission, unresolved C2, and unresolved C3 clusters for **a** cg08152411, **b** cg08888522, **c** cg13304609, **d** cg17915189, **e** cg05552874, and **f** cg02230244. These were defined as CpGs meeting all the following criteria: significant from ANOVA (*p* < 0.05/546), *p* < 0.05/411 for remission vs. unresolved C2 cluster comparison, *p* < 0.05/411 for unresolved C3 vs. C2 comparison, and cluster comparison coefficients were at least 50% different from each other

**Fig. 5:**
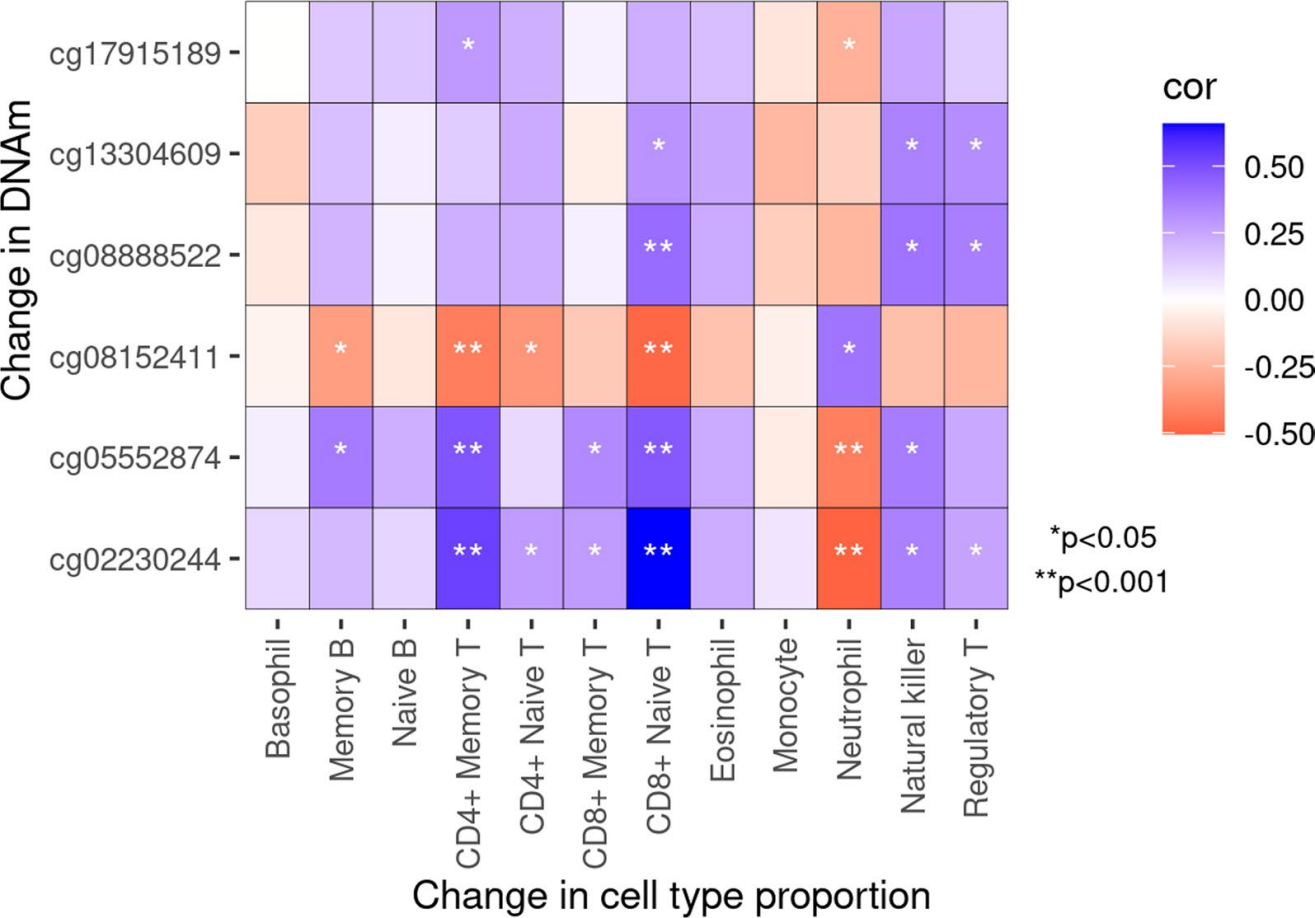
Correlation between change in cell-type proportions and change in DNAm between visits at six CpG sites that strongly differentiated patient clusters. Color represents strength and direction of Spearman correlation coefficient. P-values denoted by “*” *p* < 0.05, “**” *p* < 0.001, or empty *p* ≥ 0.05

Our second objective was to identify whether changes in DNAm might subtype patients into clinically or biologically relevant subgroups. We identified 546 CpG sites that changed between visits, agnostic of clinical outcomes (FDR *q* < 0.05 and absolute change ≥ 0.03) (Supplementary Table 4). These were significantly enriched in three Reactome pathways (FDR *q* < 0.05) involved in interferon signaling (Supplementary Fig. 1); 263 were annotated to IRGs. Using DNAm change at these sites, we identified three clusters of SLE patients (Fig. 3). All Cluster 1 patients (*n* = 12) remitted at follow-up. We called this the “remission” cluster. SLEDAI at baseline flare was similar across clusters (mean 12.3 (sd = 5.8), 10.3 (sd = 5.7), and 12.7 (sd = 5.7) for remission, cluster 2, and cluster 3, respectively) (Table 3). For clusters 2 and 3, disease was still active at follow-up (mean SLEDAI 5.3 (sd = 5.1) and 4.3 (sd = 2.8), respectively). We called these the “unresolved” C2 and C3 clusters. No clusters differed significantly by sex, race, ethnicity, or age at visits. Average age at SLE diagnosis was youngest for the unresolved C2 cluster (mean 26.9 years) and oldest for the remission cluster (mean 36.3 years). Median time between visits was largest for the remission cluster (mean 127 days) and less for the others (mean 80 days, each). Several cell-type proportions estimated from DNAm were different across clusters (Supplementary Table 5). At baseline flare, CD4 + memory T-cell proportion was highest for unresolved C2 (mean 0.08 (sd = 0.05)) and lowest for the remission cluster (mean 0.03 (sd = 0.03)) (Table 3). A similar trend was observed for CD8 + naïve T-cells. At baseline flare, average neutrophil proportion was highest for the remission cluster (mean 0.76 (sd = 0.10)) and lowest for unresolved C2 (mean 0.57 (sd = 0.14)). Medication use was similar across clusters (Supplementary Table 5). Figure 3 shows a distinct patterning of DNAm changes across clusters, particularly for the remission cluster. The remission cluster had pronounced strong changes in DNAm in the opposite direction of the weak changes observed in Clusters 2 and 3. To better characterize these patterns, we used ANOVA (global test) and linear regression (cluster-specific tests) to identify which cluster input CpGs were most strongly contributing to cluster assignments. ANOVA identified 411 input CpGs that were Bonferroni significant (Supplementary Table 6). Of these, linear regression identified 371 with significantly different DNAm change over time comparing remission to unresolved C2 clusters and 116 comparing the unresolved C3–C2 clusters (Supplementary Table 6). Each of these cluster-specific CpG sets were not significantly enriched in Reactome pathways (FDR *q* < 0.05). Both cluster-specific CpG sets included many IRGs, 43 and 41% for remission and unresolved C3 cluster comparisons, respectively. There were six CpGs that were significant in both remission and unresolved C3 comparisons (vs. C2) and had coefficients at least 50% different from each other (Fig. 4). For example, at cg08152411, within the body of *PTPRC* (CD45 antigen), there was a 15% smaller (95% CI: − 0.18, − 0.12) DNAm change between visits among the remission cluster compared to the unresolved C2 cluster but only a 6% smaller DNAm change among the unresolved C3 comparing to the C2 cluster (Fig. 4A and Supplementary Table 6). The other five cluster differentiating CpGs were within *IFIH1, IFI44L, IFIT1,* and *ENO2*. All six CpGs showed similar patterns where the remission cluster had the most DNAm change, unresolved C3 had intermediate change, and unresolved C2 individuals had the least change. Change in DNAm at these six sites were significantly correlated with change in several cell-type proportions (Fig. 5). For example, an increase in DNAm between visits at cg08152411 was correlated with a decreased proportion of memory B cells, CD4 + memory T-cells, CD4 + naïve T-cells, and CD8 + naïve T-cells and an increased proportion of neutrophils. Oppositely, increases in DNAm at cg05552874 and cg02230244 were correlated with an increased proportion of several T-cell types and decreased proportion of neutrophils. This suggests DNAm at these six sites and the identities of the methylation clusters might be driven my cell-type proportions.

**Table 3:** Characteristics of participants in SLE patient clusters identified from consensus hierarchical clustering of DNA methylation changes between baseline flare and follow-up visits

	Remission–C1	Unresolved–C2	Unresolved–C3	*p-value*
n	12	28	19	
Female, n (%)	12 (100.0)	24 (85.71)	16 (84.21)	0.36
Self-reported race, n (%)				0.27
Asian	5 (41.67)	5 (17.86)	8 (42.11)	
Black	3 (25.00)	5 (17.86)	1 (5.26)	
White	1 (8.33)	2 (7.14)	4 (21.05)	
Multiple races	1 (8.33)	1 (3.57)	1 (5.26)	
Other	0 (0.00)	2 (7.14)	0 (0.00)	
Missing	2 (16.67)	13 (46.43)	5 (26.32)	
Hispanic, n (%)	2 (16.67)	14 (50.00)	6 (31.58)	0.11
Age (years) at SLE diagnosis, mean (sd)	36.33 (18.68)	26.86 (10.98)	30.11 (11.24)	0.11
Age (years) at baseline flare visit, mean (sd)	41.83 (14.72)	36.04 (12.02)	36.68 (12.62)	0.41
Time between visits (days), median [IQR]	127.00 [83.00, 290.25]	80.00 [47.25, 276.50]	80.00 [45.50, 139.50]	0.22
Remission, n (%)	12 (100.0)	5 (17.86)	2 (10.53)	1.13E-7
SLEDAI-SELENA, mean (sd)				
Baseline flare visit	12.33 (5.82)	10.29 (5.70)	12.74 (5.68)	0.31
Follow-up visit	0.00 (0.00)	5.29 (5.08)	4.26 (2.75)	8.51E-4
Proteinuria, n (%)				
Baseline	7 (58.33)	10 (35.71)	8 (42.11)	0.42
Follow-up	0 (0.00)	5 (17.86)	1 (5.26)	0.16
Cell-type proportions, mean (sd)				
CD4 + Memory T-cells (baseline)	0.03 (0.03)	0.08 (0.05)	0.04 (0.02)	1.13E-3
CD4 + Memory T-cells (follow-up)	0.05 (0.04)	0.07 (0.05)	0.07 (0.04)	0.43
CD8 + Naive T-cells (baseline)	0.02 (0.01)	0.07 (0.04)	0.04 (0.03)	1.69E-4
CD8 + Naive T-cells (follow-up)	0.03 (0.04)	0.05 (0.04)	0.06 (0.04)	0.06
Neutrophils (baseline)	0.76 (0.10)	0.57 (0.14)	0.72 (0.10)	1.61E-5
Neutrophils (follow-up)	0.70 (0.12)	0.63 (0.15)	0.63 (0.13)	0.27

## Discussion

Using DNAm data from a multi-ethnic cohort of SLE patients with active disease, we identified 1,953 methylation sites whose change over time significantly differed depending on whether a patient fully remitted at follow-up or not. Of these, the CpG sites most differentially changed over time were located within genes relevant to immune function and autoimmunity. In addition, we identified three SLE patient clusters based on DNAm changes between visits. There were few distinct clinical features of the clusters, apart from one cluster where all members remitted. Significant DNAm changes between visits at six CpG sites, including *CD45* and *IFI* genes, were particularly different among the clusters, suggesting these may be good candidates for stratifying patients in the future. The identification of these clinically relevant clusters using DNAm data alone may help inform future disease subtyping and treatment decisions. Overall, these findings support the ability of serial DNAm profiling to uncover biologically meaningful differences beyond what conventional clinical tools can reveal.

Our study identified many CpG sites that had significantly different changes in DNAm between baseline flare and follow-up visits depending on remission status. For most of these sites, DNAm did not change for non-remitters but did change for remitters. Changes over time were upwards of 15% among remitters at 38 CpG sites. This suggests a robust effect of treatment at these sites for these 19 individuals. The most significant effect was observed for cg22873177, in the 1500 transcription start site (TSS) of *URB2.* DNAm at this site decreased 23% between visits, suggesting a shift in the chromatin state between baseline flare and follow-up from less to more permissive for translation. *URB2* is involved in ribosomal biogenesis and interacts with several proteins including interferon-inducible protein IFI16, which is a critical antiviral factor and sensor of viral DNA [34]. Autoantibodies against IFI16 have been identified in people with SLE, and a recent study showed that expression of *IFI16* was associated with SLEDAI and prognosis in lupus nephritis [35, 36].

When examining the other significantly differentially methylated CpG sites between remitters and non-remitters, most were within or near genes relevant to the immune system. For example, the second most significant effect was observed for cg14543959, in the 200TSS of *GRAMD1C*, which is associated with IL-6 levels, an important inflammatory biomarker for the SLE severity and risk of progression [37]. The third most significant CpG was within the body of *DAP*, death associated protein. A previous study found that SLE patients with the DAP1 risk allele exhibited significantly higher autoantibody titers and altered expression of immune system, autophagy, and apoptosis pathway transcripts [38]. Interestingly, a recent study found both *GRAMD1C* and *DAP1* to have significantly higher expression in SLE patients in remission compared to SLE patients with active disease [39]. Our findings do not directly compare to others because none have investigated change in DNAm over time between flare and post-flare timepoints. Another immune-related gene with a significant CpG (cg17900535) was *RASA3,* part of the RAS P21 protein activators. Ras is important for T-cell function and dysfunction has been shown to be associated with SLE [40]. Additionally, cg08399134 is within *TRMT11*, transfer RNA methyltransferase, which has been shown to selectively enhance protein translation to drive T-cell proliferation in vivo when methylated and is associated with lupus nephritis [41, 42].

Results from consensus hierarchical clustering of DNAm changes identified three SLE patient clusters. Interestingly, a remission cluster emerged, despite solely relying on DNAm data for clustering. It’s important to note that our sample had 19 people fully remit over the follow-up period, but only 12 were classified into the “remission” cluster. This highlights that, while the clusters were clinically relevant, they did not completely correspond to clinical features. The two “unresolved” disease activity clusters only differed in proportion of neutrophils at the baseline flare visit (57% for C2 and 72% for C3 clusters). Neutrophil proportions were similar for these clusters at follow-up. Neutrophils have been implicated in SLE pathogenesis and organ damage [43].

An important finding from our cluster analysis was that, at six CpG sites, the average DNAm change between visits was distinct for each cluster. For example, at cg08888522, within the body of *IFIH1*, DNAm increased, on average, from flare to follow-up visit 25% for the remission cluster, 13% for the C3 unresolved cluster, and only 1% for the unresolved C2 cluster. *IFIH1* senses double-stranded RNA and activates type I interferon signaling [44]. Several studies have found an association between *IFIH1* and SLE, including a de novo rare gain-of-function genetic variant identified in a severe SLE patient and several common variants associated with SLE susceptibility, IL-18 and granzyme B serum levels, and autoantibodies in SLE patients [45–48]. Another “cluster differentiating CpG” included cg13304609 in the TSS1500 of *IFI44L*. Methylation in the promoter of *IFI44L* has been proposed as a diagnostic biomarker for SLE [49, 50]. Additionally, cg08152411, within the body of *PTPRC* (CD45 antigen), was a “cluster differentiating CpG”. Autoantibodies to CD45 have been found in SLE [51, 52]. Our results also showed these methylation changes were correlated with changes in SLE-relevant cell-type proportions. This suggests that a shift in immune cell composition is reflected in change in DNAm in immune relevant genes. Single cell genomics approaches should be used to determine the relevance of this correlation to better understand disease heterogeneity. Altogether, this cluster analysis showcases the heterogeneity of the immune response of SLE and indicates that DNAm data may be useful for subtyping SLE patients who cannot be distinguished from each other using clinical data alone.

This study has several strengths and limitations. The majority of SLE studies are cross-sectional and have patients with relatively low disease activity. Our study has two timepoints and only includes individuals actively experiencing a physician-diagnosed flare and warranting a treatment change at study enrollment. This sheds light on mechanisms underpinning active disease, which might be missed in other studies. Our sample is also ethnically and racially diverse. SLE patients from non-European populations, such as Hispanics, African Americans, and Asians, develop SLE at a younger age and experience worse disease manifestations than patients of European descent. Yet, these groups are underrepresented in translational studies of SLE. Despite having a highly informative set of individuals, our sample size was modest (118 samples). This is partly due to the challenge of recruiting and studying active SLE flaring patients in clinics. Still, we were able to find many CpG sites where DNAm changed differentially depending on remission status and identify meaningful patient clusters. Additionally, our study involved a real-world uncontrolled medication setting, making it difficult to study treatment response. While we adjusted for medications using MCA, we may not have captured differences in medication use such as adherence, dosage, or combinations. Patients also had different follow-up times depending on when they were seen at outpatient visits. This is relevant for medications that take longer to garner an immunological response. Ideally, in the future, DNAm would be profiled longitudinally in clinical trials for prediction of treatment response and identification of mechanistic pathways underlying response. This would also improve our ability to determine whether serologically active, clinically quiescent patients have different patterns of DNAm change compared to those whose remission included serological inactivity. Finally, we were not able to replicate our findings in an independent cohort because, to the best of our knowledge, such longitudinal, repeated sampling of DNAm among active SLE cohorts does not exist. However, we did use consensus hierarchical clustering, which conducts clustering 1000 times with slightly different resampling of patients and data, to build confidence in the replicability of findings.

## Conclusions

Changes in DNAm during active SLE were associated with remission status and identified subgroups of SLE patients with several distinct clinical and biological characteristics. Knowledge of DNAm patterns might help better inform SLE subtypes, leading to targeted therapies based on relevant underlying biological pathways. Further studies are needed to elucidate the potential importance of the associated CpG sites and genes in immune function and to validate the prognostic or diagnostic value of the individual CpGs or panels. Additionally, another longitudinal timepoint would be useful for identifying whether changes in DNAm might predict future disease status and damage.

## Supplementary Information

Supplementary file 1

Supplementary file 2

## Data Availability

Data is available upon request.
