# Supplementary material for: Changes in DNA methylation are associated with systemic lupus erythematosus flare remission and clinical subtypes
Source: Clin Epigenetics. 2024 Dec 18;16:181. doi: 10.1186/s13148-024-01792-x (PMC11656870; doi:10.1186/s13148-024-01792-x)
Supplement: Supplementary file 1 [file 13148_2024_1792_MOESM1_ESM.docx]

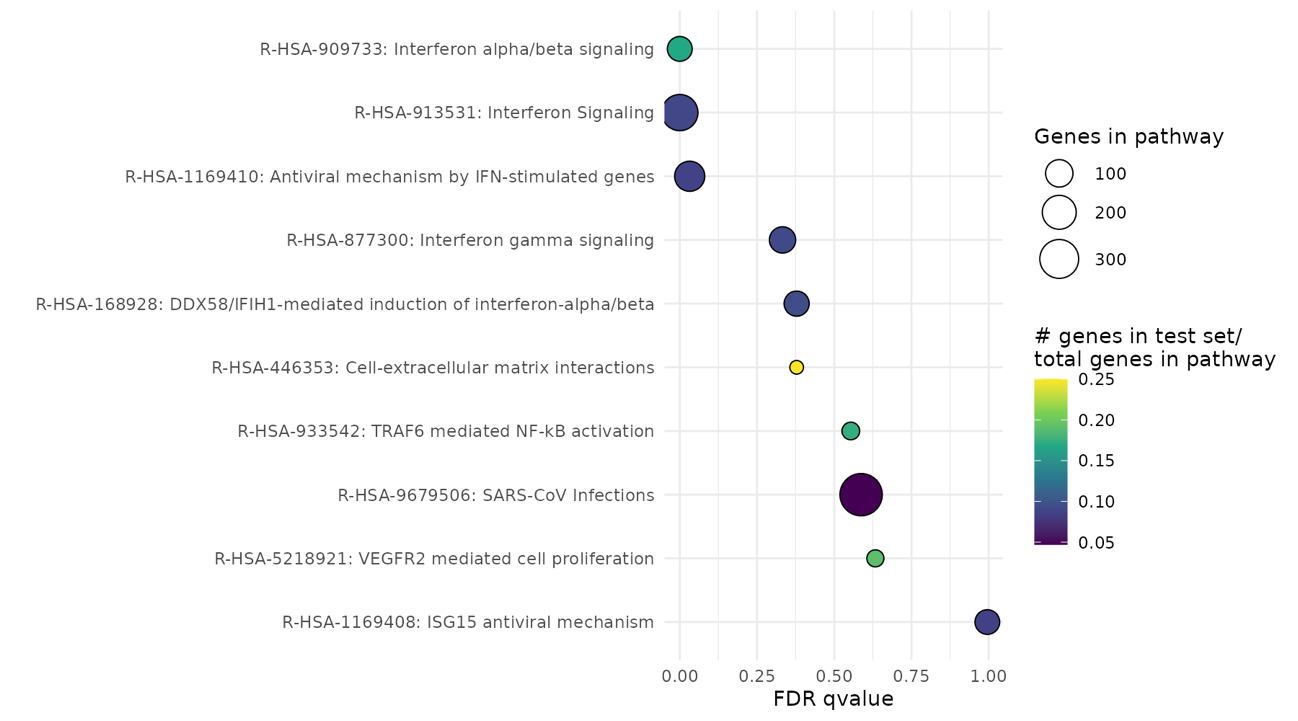


**Supplementary Figure 1.** Reactome pathway analysis results for 546 CpGs used for consensus hierarchical clustering.
